# ADMIRE: analysis of digitalized human-machine interactions and relations–looking closer at the tacit dimensions of human-machine relations as part of integrated research

**DOI:** 10.3389/frobt.2026.1747442

**Published:** 2026-02-18

**Authors:** Arne Manzeschke, Galia Assadi, Jochen J. Steil, Sonja Spörl

**Affiliations:** 1 Lutheran University for Applied Science Nuremberg, Nuremberg, Germany; 2 TU Braunschweig, Brunswick, Germany; 3 Center for Logic and Philosophy of Science, KU Leuven, Leuven, Belgium

**Keywords:** anthropology, human-machine interaction, human-machine relations, intelligent systems, robotics

## Abstract

ADMIRE (Analyzing Digitalized Human-Machine Interactions and Relationships) is a tool that was developed and tested as part of the Integrated Research Cluster. Its aim is to make explicit the implicit assumptions about humans and machines, as well as their potential and limitations. In this way, it provides a basis for structured, reflective research and development processes relating to human-machine interactions, as well as providing a starting point for ethical considerations in technology design. This article outlines the initial research and development approach and the insights gained from various research projects and application settings. We then trace this back to anthropology and the implicit images of humans and machines that determine the processes of research and development, and often prevent the implementation of ‘technological solutions' to social problems. Here, we introduce the ADMIRE tool, along with its theoretical background and practical deployment. Finally, we reflect on the limitations of the tool itself and our experience to date.

## 1 Introduction

The idea that technology as such is a neutral thing and that it is up to society to decide whether it should be used for the good has long since been abandoned. The German engineer Hans Sachsse already signaled this problem at the beginning of the 1970s: “We have grown up convinced that technical progress is a good thing in every respect and under all circumstances. I don’t think we can say that unreservedly today. In recent decades, technical progress has taken on new forms, at first imperceptibly, but then at an increasing pace. Nuclear energy, information technology, genetics, biotechnology and pharmacology are exposing us to new risks of progress and we are currently experiencing a revolution in our entire living conditions in the public and private spheres.” (Sachsse, 1993).

Some 50 years later, the topics of synthetic biology, genome editing and artificial intelligence need to be added. Building on the developments described by Sachsse, these topics are contributing to a radical transformation of our individual and societal life. Radical is meant in a literal sense that humans now are able to modify the biological basis of their own existence to trans- or post-humans (Loh, 2023). At the same time, we are going to reconstruct the biology of other living beings on a technological basis. Thirdly, we are constructing technical entities that interact with us human beings in a way very close to our social life, although their legal, moral and social status is still completely unclear.

We are not dealing with one technology in isolation, but with the convergence of many multifaceted, intertwined technologies, which increases the complexity of what we call “challenges” or “problems”. This issue has been discussed for over 20 years under the umbrella of *converging technologies* (NBIC = Nano-, Bio-, Information Sciences and applied Cognitive Science; cf. Roco and Bainbridge, 2002; critically: Büscher, 2009). Recently, Co-founder of DeepMind and Infliction AI, Mustafa Suleyman has argued for the *containment* of “the coming wave” Suleyman and Bhaskar (2023) which is emerging from current technologies, especially related to artificial intelligence and synthetic biology. While these technologies will set free enormous potential for humanity, they will confront us at the same time with unprecedented risks concerning the living condition of more than our own species. It would be short-sighted to address these challenges from only a technical perspective. The contemporary philosopher Shannon Vallor underpins this: ‘We are technomoral creatures to the core; that is, we allow and have always allowed the things we make to reshape us. The only question is whether this process is deliberate and wise or unreflective and reckless.’ (Vallor, 2015) How would a deliberate and wise process look like? This is not just a question of technology. All the more, it is also a normative question: How do we want to live, to work, to travel, to consume and so forth? How should the framework for public order look like? (cf. Winner, 1980, 128f.) This article will contribute to this very fundamental questions with an interdisciplinary approach and focus on human-machine-interaction. In a first step, we place ADMIRE (*A*nalyse *Digitalisierter M*ensch-*M*aschine *I*nteraktionen and *RE*lationen; i.e., *A*nalysis of *D*igitalized Human-*M*achine *I*nteractions and *RE*lations), the model which we present in this article, in the broader context of research programs and methods. We show that the idea of incorporating non-technical perspectives into technology research and development has become widely accepted in recent decades. In a second step, we outline some theoretical references of ADMIRE in order to present the model itself and how to work with it in a third step. Finally, we discuss strengths, limitations and further steps.

## 2 The demand for integrative R&D methods

The interplay between human beings and highly sophisticated technical systems is expressed using different terms, but these cannot conceal at all the embarrassment about the very fundamental question: Who or what is this system with which we interact, interplay, cooperate, co-act, or what the more terms there may be. Despite the fact that we do interact with technical systems, we still do not fully understand who or what we are dealing with. Is it merely a programmable apparatus with some more features than a ticket vending machine? Should electronic personhood be ascribed to it, as the European Parliament suggested some years ago—while there were no Large Language Models at all? Should robots have standing (Gerdes et al., 2022)? Should they even be granted citizenship, as happened with Hanson Robotics’ Sophia?

Undoubtedly, the performance of these technical systems which are equipped with artificial intelligence and operate with context-sensitivity and high precision manipulation sets new standards for human-machine-interaction. This does not only apply to the industrial sector, but also to everyday life, where conversational capabilities, emotion recognition and expression give rise to a new social counterpart for us human beings (e.g., Lugrin et al., 2021). Bringing these “ever more sophisticated robots and other manifestations of artificial intelligence” into play, poses serious questions for all sectors of human life and beyond: ‘Humankind stands on the threshold of an era when ever more sophisticated robots, bots, androids and other manifestations of artificial intelligence (AI) seem to be poised to unleash a new industrial revolution, which is likely to leave no stratum of society untouched. The development of robotics and artificial intelligence raises legal and ethical issues that require a prompt intervention at EU level.’ (EU, 2015). Three years later, the EU Parliament delivered a respective resolution: “Civil Law Rules in Robotics” (EU, 2018). In addition to the legal regulation of technology, the social, ethical, anthropological and psychological questions must also be addressed. On the one hand, this requires disciplinary research in the aforementioned fields, and probably some more, but it also necessitates the integration of the various perspectives to design the research, development, application and disposal of these technical systems in a manner that is ultimately people-friendly, beneficial to life as a whole and sustainable. The great challenge we face is underpinned by the fact that the technology we introduce will determine who we will be in the future. Our decisions regarding technological research and development largely determine who and how we will be as human beings.

If you want to design technical systems with this requirement in mind, then an enormous amount of knowledge and methods is necessary in order to succeed. When reflecting on the forms of expertise needed, we often consider explicit forms of knowledge originating from different disciplinary backgrounds. Research modes like *Responsible Research and Innovation* (RRI; cf. Hoven et al., 2014), *Value-Sensitive Design* (cf. Hoven et al., 2015; Friedman and Hendry, 2019; Friedman et al., 2017) and *ELSI* (i.e., Ethical, Legal and Social Implications; cf. Myskja et al., 2014; ELSI-Hub, 2025) stress the importance of broadening the scope by considering non-technical perspectives equally relevant for successful research and development. By including the reflection of non-technical preconditions and effects of technological systems, an important step towards a more integrated form of technological construction was made. This insight has been essentially considered by the most important reports and frameworks on autonomous and intelligent systems, e.g., *Ethically aligned Design* by IEEE (2017), or *Ethics Guidelines for Trustworthy AI* by the High Level Expert Group on AI set up by the European Commission (2019), followed by the EU AI-Act (2024) (cf. Djeffal, 2026; Manzeschke, 2026). However, these research modes often focus on explicit forms of knowledge, which can be detected in codes of law or by conducting surveys, but simultaneously underestimate the meaning of implicit/tacit forms of knowledge/skills, which was already tackled very early by Polanyi (1966) and has been scrutinized and elaborated since then (cf. e.g., Reber, 1996; Pozzali, 2007; Thornton, 2023).

The Integrated Research program is part of a series of demands relating to funding policy and research practice that aim to minimize the undesirable side effects of technical innovations on social life. It was launched by the Federal Ministry of Research, Technology and Space (BMFTR, formerly BMBF) and offers researchers and developers a platform to test and discuss methodological and theoretical approaches in the broad field of human-technology relations. (BMBF, 2025). As part of this program, we have developed ADMIRE with the aim of making these implicit forms of knowledge or implicit assumptions about humans and technology explicit and thus contributing to a more reflexive technology development.

Dealing with implicit/tacit knowledge/skills in the R&D-Process of robots, two layers have to be distinguished: 1) implicit presuppositions about the needs, potentials and limitations of human beings and the potential of robots to answer these (cf. Björn et al., 2020) 2) implicit/tacit knowledge/skills of human beings that shall be implemented in robots (cf. Tatiya et al., 2023). We mainly deal with the first layer in the following presentation.

The reflected objectives, models and rules that consciously and explicitly guide the development process of technical systems are complemented by implicit, unconscious assumptions about users, interaction and the relationship between humans and technology, which are unreflected but not ineffective. Both conscious and unconscious ideas guide technological development and usage processes by orientating people’s actions, thoughts and feelings and thus influencing the interpretation of the interaction situation and the selection of a suitable behavior. These implicit ideas about humans (conceptions of humans–in German: “Menschenbilder”; cf. Zichy, 2021) and technology (technical imaginaries–in German: “Technikbilder”; cf. Huber, 1989) therefore indirectly influence the research and development of human-machine relations—and, furthermore, the interactions between users and the developed technology and thus determine its success or failure. In a broader sense and beyond considering concrete interactions only, these ideas also affect technology in its mediality (Hubig et al., 2013) and gradually change the interacting individuals’ relationship to themselves. The same applies *vice versa*: the deployment of technology impacts on the human’s perception of interaction, why it is necessary and how it should work. In order to avoid mismatches between the implicit ideas of developing engineers and users, which can lead to failure regarding implementation, it is necessary to reflect systematically and explicitly upon these implicit conceptions.

In addition to this basic idea, we pay close attention to the narratives (e.g., Cave et al., 2020; Cave et al., 2023) that inform our understanding of intelligent machines and their respective R&D processes. We are especially interested in the language used to communicate about them. Metaphors play a central role here, which can be explained by the fact that new technical developments cannot yet be described using proper terms. Instead, similarities, analogies and comparisons with familiar concepts are used to facilitate understanding and appropriate handling (Gransche et al., 2026; Gransche and Manzeschke, 2020; Manzeschke et al., 2024; Barnden and Lee, 2001; Ulrich, 2026). From an anthropological perspective, the entire process of metaphorization is also due to the fact that we do not always have theoretically sound evidence for the phenomena we are dealing with. If we had this evidence, we would not need to ‘beat about the bush’. However, this is precisely what metaphor does; it reflects our relationship with reality. As Blumenberg pointedly put it, this relationship is ‘indirect, circuitous, delayed, selective and, above all, “metaphorical”,’ (Blumenberg, 1971, 115; transl. AM).

Another crucial issue for our model is of anthropological and psychological nature. Emotions play a very significant role in the process of construction of human-machine-relations. They do it on two levels: 1) as part of the envisaged relationship or interaction between human and machine, 2) as part of the process of advising on the design and marketing of the product. With our model we very much concentrate on level 1) and prefer a more nuanced approach to human emotions (cf. Feldman et al., 2019) than so-called “Facial Activity Coding System” (FACS), originally suggested by Ekman and Friesen (1978). Lately, we have merged an anthropological perspective, inspired by the “New Phenomenology” (cf. Schmitz, 2014; 2019), with a technological, sensor-based approach to argue for a thorough investigation of human emotions and their technical representations in human-machine-relations, while this goes to the very heart of human self-understanding (Assadi et al., 2026).

Last but not least, our model implies necessary lessons to be learned. Technological progress should be accompanied by a societal anchored process in which we learn what this new technology is all about and how we should deal with it. That is by no means a matter of cause. Learning *about* the new technology goes hand in hand with learning *with* the new technology. These closely interlinked learning processes increase the complexity and the demands placed on us (cf. Stracke, 2026). Above all, however, these efforts require a moral compass. In this sense, learning means improving the knowledge, sharpening our judgement, and constantly reflecting on serious moral questions, as philosopher of technology, Gernot Böhme, once put it: Ethics is the reflection and examination of serious moral questions, and “serious moral questions are those that determine who and what we are as human beings” and “in what kind of society we will to live together as human beings”. (cf. Böhme, 1997, 17). This, roughly sketched program has certain affinity with the idea of Drigas et al. (2022) on meta-learning with smart technologies. Among other things, this requires an intensive examination of the concept of intelligence, which is used equally for living beings and technical systems (cf. Manzeschke and Gransche, 2025). Based on this we would like to stir our further considerations more in the direction of politics.

## 3 ADMIRE–An Analytic Tool and its methodology

The technical advances we have been talking about here are changing human living conditions—and the living conditions of other beings—so fundamentally that any further technical developments must be conceived and tested alongside anthropological, ethical, psychological, social, and political considerations (cf. Crawford, 2021; Brook in interview with‚ ”Godfather of AI”, Geoffrey Hinton, Brook, 2023). Doing so, needs systematic and thorough scrutiny of those images of human beings and technology guiding the process of invention and construction implicitly or explicitly.

Among the various technological entities, robots that are designed to interact with humans have a specifically strong influence on social processes and are therefore of particular ethical interest. We will present ADMIRE, an analytical tool for Digitalized Human-Machine Interactions and Relations, its theoretical background, its methodology to provide an instrument for structured reflection on the implicit effects that interaction design has on shaping our relationship to digital interaction technology.

We start from the observation that many projects in the field of human-machine-interaction pay little attention to the images of humans on the one hand and technology on the other that guide their research and development. Even though these images are overlooked, they can have a significant impact on processes and products, often for the worse.

In order to cope with potential mismatches before and during the development process, it is necessary to explicate these implicit conceptions of humans and technical imaginaries. Various schemes have been developed to describe relationships between humans and machines in a differentiated way, such as the classification according to automation levels (Steckhan et al., 2022), the taxonomies of autonomous systems with ‘degrees of autonomy’ (Onnasch and Roesler, 2021), or the “degrees of collaboration” in robotics (Beer et al., 2014), which are often used in the technical field. Coeckelbergh argues for a methodological turn in robot-ethics to an “ethics of appearance and to an ethics of human good understood as emerging in experience and practice” (2009, 219). In contrast to him we refrain from an ethical point of view at this point, limiting our efforts to an anthropological description. With our model we refer to the AMTIR heuristic developed by Gransche et al. (2014), which can be used to distinguish between different types of relations: *Use* (use of tools, no delegation of control), *Operation* (operation of machines, delegation of operational control/steering), *Interaction* (delegation of strategic and operational control, negotiation of target priorities) and *Coaction* (acting in technical systems). The relationship can then be categorized according to these types. These different types of relationship can in turn be linked to the roles that occur within them. It allows to reflect these assumptions against the background of the development and implementation context of the technology and their concrete effects on human-machine interaction as well as the human-machine relationship to be shown.

ADMIRE is intended to enable a structured reflection process for all those involved in R&D. It has a low barrier on methodological prerequisites and does not require specific prior disciplinary knowledge. It helps clarifying the (pre-)orientations regarding humans and machines that are introduced into the development process by means of explicating them. Human-machine interaction cannot only be understood in its current situatedness and thus from the present, but it also depends on previous orientations in the sense of assumptions, judgments and behavioral patterns about human-technology relationships that were acquired in earlier times (Stegmaier, 2008, esp. ch. 6). The acquired orientation influences the perception, the emotional and rational interpretation of the situation as well as the behavior in the concrete interaction situation and can only be identified through targeted analysis. This form of analysis is currently not carried out systematically within the framework of technology development projects, as these usually have a selective focus on the interaction situations to be constructed. Usually they are considered in isolation from their historical, social and cultural background and their preconditions. In addition to an orientation function, ADMIRE also offers the potential to recognize and avoid inadmissible, discursive fallacies through the act of reflection. It is to be understood as a tool for gaining knowledge, i.e., its methodology does not prescribe normative states, but is rather descriptive of the human-technology relationship. With regard to integrated technology development, ADMIRE offers the options of both (1) reconstructing *ex post* conceptions of humans and machines by those who developed existing technical systems, and (2) identifying and constructively addressing the corresponding implications *ex ante* as part of the planning and implementation of projects. The effects of conceptions of humans and machines on interaction partners, technical systems and the human-technology relationship, are thus accessible to both designers and third parties.

**FIGURE 1:**
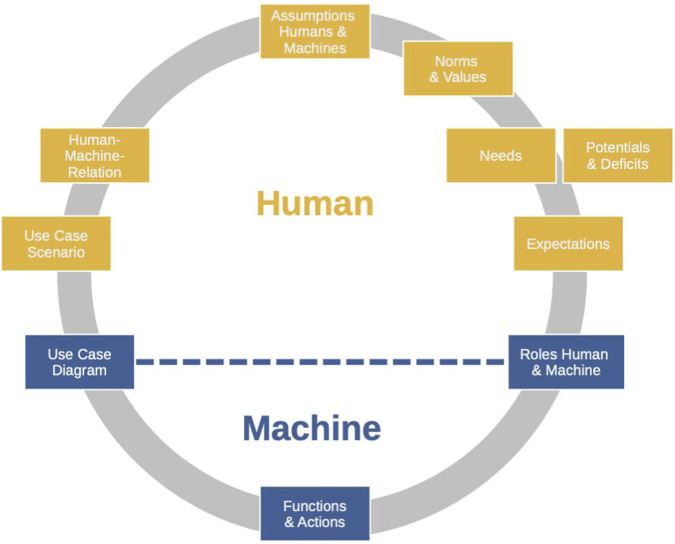
ADMIRE–An Analytic Tool for orientation in digitalized living environments; Source: author’s own compilation.

ADMIRE (see Figure 1) is easily accessible in both the technical and non-technical areas by making use of a central concept of technology development, the so-called *use-case diagram*. This is a graphical tool in software and technology development to represent the interactions of a technical system with its users in a structured manner, where these users can be humans or other technical systems (Kecher, 2006). The use-case diagram is easy to understand even without prior technical knowledge, it should be known to those with (software) technical training as part of the standard UML (Unified Modeling Language), and it is ideally placed at the interface between human and machine in order to enable the professional-cultural transition between the spheres of non-technical reflection on the preconditions of interactions and the technical realization of concrete artefacts. It is, thereby, well suited to operationalize a structured reflection about the implicit preconditions of technological development in a way that is easily accessible to all those involved and is linked to the specific technical system.

The proposed analytical tool is applied in form of a guided workshop with developers and system designers. In the course of the workshop, the “boxes” in Figure 1 are discussed at hand of respective structured questions in order to explicate the implicit conceptions about humans and machine. In the following we describe the different steps in a rather general manner.

The starting point of the analysis tool is the *Human-Machine Relation*. This relation is examined and described in more detail using some theory-based constructs that are useful for understanding the interaction between humans and technology. After the relationship has been examined against the background of these constructs, it is possible to derive a clearer understanding of images of humans and machines that have an effect on the human-machine relationship. The human-machine relationship is put in concrete terms in the *Use-Case Scenario* and can therefore be analyzed further. The latter explains the application context of the technology to be developed as well as the intended objective of the human-technology interaction. Such a scenario is usually required in project and research proposals, which is why it offers a low-threshold and connectable concretization for reflecting on and defining the human-machine relationship. The Use-Case Scenario enables the transition to the technical level, which is substantiated in the form of a *Use-Case Diagram*. It contains a formal description of how the technology works in relation to various actors, including human beings. *Functions, Actions* and their hierarchies are defined here. The *Use-Case Diagram* also indicates which roles the various actors have in the human-machine relationship and whether and which interactions occur between them.

The *Use-Case Diagram* is compatible with the social science and philosophical construct of complementary *Roles of Humans and Machines*, the exercise of which in a specific interaction situation reduces complexity, creates certainty of expectations and actions, stabilizes order and thus offers orientation. On the human side, roles are linked to behavioral expectations, patterns of thinking and action, thoughts and feelings. By referring to (social and technical) roles, expectations of the behavior, feelings and thoughts of humans and the behavior of machines can be described, which creates a practical approach to render abstract and universalizing images of humans and machines concrete and analyzable. This concretization allows elements of the human and machine conception as well as the relationship between the two to be analyzed.

Roles and the expectations connected to roles can be linked to the *Potentials and Needs* of the role holders. In addition to potentials, deficits associated with the respective assigned role can also be derived. The potentials and deficits that are linked to the roles of human beings and machines in turn allow conclusions to be drawn about the *Norms* that are linked to them and articulated in them. Behavioral Norms formulate the rules that are linked to holding a role and in turn articulate and refer to abstract *Values* (such as freedom, justice, equality, etc.). Access via norms and values also enables reflection on the context of the respective interaction, since both norms and values, always refer to a specific historical and social scope. Furthermore, roles and the patterns of interpretation and action associated with them, as well as expectations, can be understood from a psychological and anthropological perspective not only as normative constructs in the service of reducing complexity and providing orientation, but also as a socially established and legitimate form of response to human needs. Human beings can therefore be understood not only as those that can be characterized by potentials (e.g., autonomy) and deficits (e.g., disability) but also as beings that are characterized by *needs*. Structurally analogous to the discussion of human needs, functional requirements can be identified on the technical side, the fulfillment of which must be ensured for the interaction to succeed. Depending on the type and amount of needs satisfied by the roles in the human-machine relationship, different values can then be inferred.

With the help of the identified norms and values associated with human beings and machines respectively, we can finally ask how people are perceived in human-machine relationships, and whether this perception is what we are aiming for. The answers articulate possible conceptions of human beings within these relationships, providing a basis for further reflection.

## 4 Working with ADMIRE

The methodological approach consists in constructing and using a tool that contains various elements that build on one another but can also be considered separately. Abductions in a formal logical sense are used to draw conclusions from one element to another. Abductions are functional for ADMIRE, even if they are not strictly speaking logically valid. Due to the implicit and unconscious nature of conceptions of humans and machines and the fact that they have (so far) mostly been lacking in analysis, it is impossible to have the conceptions that are actually effective in the construction available, or to reconstruct them *ex post* deductively. The direction in which the analytic instrument is run through, as well as the number of elements to be considered in it, is not conclusively determined. However, the probability of discussion fallacies or false conclusions increases the more elements of the analytic tool are not considered. Extensions or modifications are always conceivable and feasible, which means that the tool can be adapted to the respective context. Interested research teams or development projects can ask for a workshop (ADMIRE, 2025). This is then tailored to the specific needs and starting conditions. So far, workshops have been offered only in German; a workshop in English is a matter of negotiation.

We have tested the tool in four different settings so far, i.e., two ex ante scenarios where the technical product is to be constructed from the scratch respectively where it is close to the market launch. Two ex post scenarios where the users wanted to reassure their design decisions. In all settings, we looked for the starting point in the desired scenarios: What benefit should be achieved by using human-machine interaction and what problem should be addressed? So the U*se-Case Scenario* formed the entry point for a detailed discussion fueled and structured by impulse questions, e.g.,: Should the system fulfil a social objective and if so, which one? What reaction should the system show to certain human actions? What reaction(s) is the human being expected to show? Which should they show? Are there reactions that are undesirable? What reaction does the technical system show?

As the team moves through the boxes of the circle, they gain a clearer understanding of the implications of their own design plans. They may come across contradictions in their own expectations or confront themselves with their moral reservations.

Furthermore, we ran workshops with clients from a variety of backgrounds, including software engineers, interdisciplinary research groups, and nursing and social work practitioners. While they all worked well with the tool, they needed different guidance regarding the terminology. For example, engineers immediately understood the meaning of a Use Case Diagram, whereas practitioners and those unfamiliar with engineering did not. Based on our limited experience to date with the tool, we can conclude that it is effective in different settings, but requires careful, client-oriented preparation.

## 5 Conclusion

The analysis tool was evaluated by relevant experts and validated in a small number of initial test workshops. Our initial experience suggests that the analysis of implicit images of humans and machines has succeeded in establishing a more differentiated and accurate understanding of the human-machine relationship. On the other hand, it enables a new connection between technological and anthropological reflection and ethical evaluation. This enriches the technical perspective with non-technical aspects and makes the anthropological foundations of ethical judgements identifiable and accessible for reflection and deliberation. Thus, the analysis tool addresses a gap in the connection between technical and anthropological expertise (cf. Manzeschke and Steil, 2024), as well as in the connection between anthropological and ethical reflection.

## Data Availability

The original contributions presented in the study are included in the article/supplementary material, further inquiries can be directed to the corresponding author.
